# Nanoparticle albumin-bound paclitaxel in a patient with locally advanced breast cancer and taxane-induced skin toxicity: a case report

**DOI:** 10.1186/1752-1947-8-6

**Published:** 2014-01-03

**Authors:** Beatriz Cirauqui Cirauqui, Vanesa Quiroga García, Clara Lezcano Rubio, Maria Iciar Pascual Miguel, Laia Capdevila Riera, Nuria Pardo Aranda, Sara Vizcaya Martín, Antonio Mariscal Martínez, Clara Rodríguez Caruncho, Mireia Margelí Vila

**Affiliations:** 1 Department of Medical Oncology, Catalonian Institute of Oncology, Germans Trias i Pujol Hospital, Badalona, Spain; 2 Department of Pharmacy, Catalonian Institute of Oncology, Germans Trias i Pujol Hospital, Badalona, Spain; 3 Breast Pathology Unit, Germans Trias i Pujol Hospital, Badalona, Spain; 4 Department of Dermatology, Germans Trias i Pujol Hospital, Badalona, Spain; 5 Catalonian Institute of Oncology. Germans Trias I Pujol Hospital, Carretera de Canyet s/n. 08916, Badalona, Spain

**Keywords:** Breast cancer, Nab-paclitaxel, Taxane-induced toxicity

## Abstract

**Introduction:**

Taxanes have demonstrated effectiveness in the treatment of breast cancer, the most common type of cancer in women. The toxicity profile of taxanes (including skin toxicities) induces dose adjustment, delay, or discontinuation, which prevents a sufficient dose intensity to achieve a response. Nanoparticle albumin-bound paclitaxel, a solvent-free form of paclitaxel, prevents toxicities and reduces the pharmacokinetic interferences between paclitaxel and other drugs.

**Case presentation:**

We describe the case of a 55-year-old Caucasian woman with locally advanced breast cancer treated with neoadjuvant therapy who developed secondary skin toxicity due to delayed hypersensitivity to taxanes. She received Adriamycin® (doxorubicin), cyclophosphamide and docetaxel and developed toxicity that promoted treatment delay and a switch to weekly paclitaxel. After the third and fourth weeks of treatment, paclitaxel toxicities also induced treatment delay and paclitaxel was switched to nanoparticle albumin-bound paclitaxel. She completed the five planned nanoparticle albumin-bound paclitaxel cycles with acceptable tolerability (including persistent grade 2 neuropathy) and without dose delay or adjustments. Clinical response was achieved although pathological response was not good.

**Conclusions:**

Nanoparticle albumin-bound paclitaxel treatment is a good option for patients with breast cancer with taxanes-related skin toxicity. This drug allows the treatment to be completed with acceptable tolerance in our case.

## Introduction

Breast cancer is the most common type of cancer diagnosed in women [1]. Breast cancer is a heterogeneous disease regarding gene expression, morphology, clinical course and treatment response [2].

Taxanes have shown significant activity in early and advanced breast cancer [3]. Due to the hydrophobic properties of taxanes, solvents are required for intravenous administration (Tween 80® and Cremophor®) [4]. Solvents limit the clinical effectiveness of taxanes, induce a toxic response [2] and increase the adverse effects experienced by patients with breast cancer, such as myelosuppression, neurotoxicity, arthralgia and/or myalgia, and hypersensitivity reactions. It has been shown that Cremophor® used as a solvent in paclitaxel is associated with major side effects including neutropenia, hypersensitivity reactions, and neuropathy due to axonal degeneration. Tween 80®, the solvent used in docetaxel, has been shown to partially contribute to fluid retention by altering membrane fluidity. These toxicities often require dose delay, adjustment or even discontinuation of taxanes.

Nanoparticle albumin-bound paclitaxel (nab-paclitaxel; Abraxane®) is a solvent-free form of paclitaxel, which eliminates the risk of toxic solvents. Substitution of solvent-based paclitaxel prevents toxicities such as hypersensitivity reactions and long-standing neuropathy, reduces the pharmacokinetic interferences between paclitaxel and other drugs, and decreases the complexity and inconveniences of paclitaxel dosing [5].

We here discuss a case of a patient who received taxanes and developed skin toxicity; the taxanes were replaced by nab-paclitaxel, which allowed her to complete her neoadjuvant chemotherapy schedule.

## Case presentation

A 55-year-old Caucasian postmenopausal woman with a history of penicillin allergy, dyslipidemia with hypolipidemic diet treatment and no substance abuse, was referred to our Breast Pathology Unit when a left axillary lymph node was noted on palpation.

The examination revealed a hard fixed matted lymph node mass of 5×3.5cm on her left axilla, and a 1×1cm lump in her left breast axillary tail. The mammography showed a 20 to 24mm, dense, well-defined left breast nodule (upper external quadrant), and dense well-defined nodules in the left axillary space, the largest one measuring 4 to 5cm. In addition, an ultrasound scan revealed a 2cm well-defined, hypo to anechoic nodule in the upper outer quadrant of her left breast tail. The left axillary nodules were abnormally enlarged lymph nodes, all of them hypoechoic with cortical thickening. Eccentric hilum was absent in some of the nodes.

The examination was completed with a magnetic resonance imaging (MRI) of her breasts, which detected a lesion at the junction of the outer quadrants, close to the retroareolar region of her left breast. The lesion had undefined margins and two adjacent areas of focal uptake measuring 2×1.7×2cm, and multiple enlarged left axillary lymph nodes extending from her breast tail to levels I and II.

A biopsy of the retroareolar area was consistent with infiltrating lobular carcinoma, 100% of the tumor cells were positive for estrogen receptor, negative for progesterone receptors (PR) and without overexpression of c-erbB-2 (score 1).

A needle biopsy from two of the enlarged axillary lymph nodes and the enlarged intramammary lymph node in her breast tail was consistent with metastasis of carcinoma.

The staging evaluation showed no evidence of distant spread.

Neoadjuvant chemotherapy was administered based on the high burden of axillary disease and the risk of microscopic dissemination, and on the hormone status (PR negative) that suggested a luminal B tumor type which is likely to benefit from chemotherapy and evidence of response after neoadjuvant chemotherapy in lobular-type tumor [6]. The patient received four cycles of Adriamycin® (doxorubicin, 60mg/m^2^) and cyclophosphamide (600mg/m^2^) every 3 weeks, achieving partial response on physical examination. Docetaxel (100mg/m^2^) was started subsequently, with cycles every 21 days for 4 planned courses.

**Figure 1:**
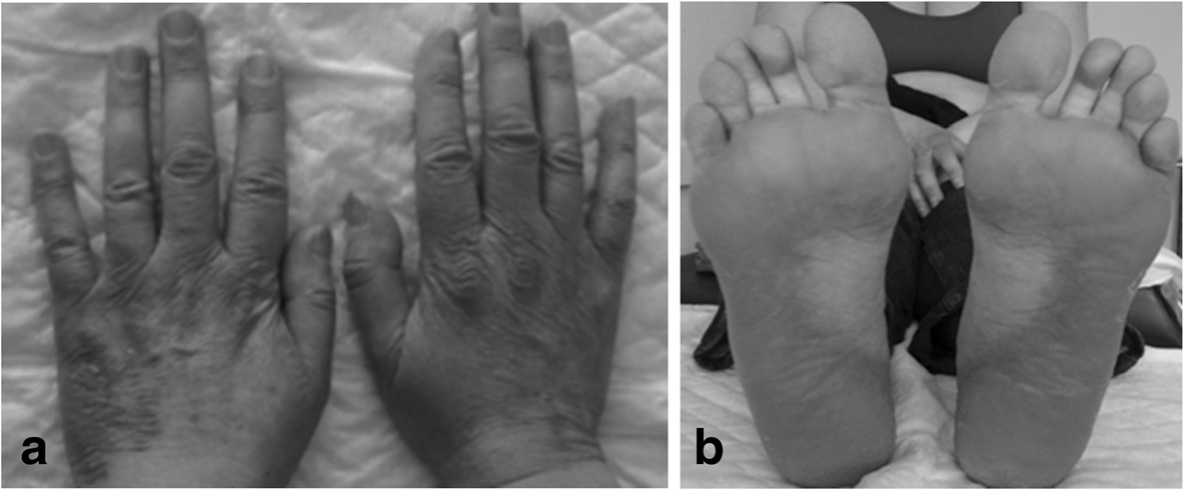
Grade 3 skin toxicity secondary to docetaxel.

After cycle 1, the patient developed grade 3 skin toxicity (with a predominance of erythrodysesthesia on the soles of her feet and on the palms and dorsa of her hands), grade 3 arthralgia and myalgia, and grade 2 asthenia (Figure 1). This significant toxicity promoted a 1-week chemotherapy delay. Symptoms were managed with anti-inflammatory drugs and oral and topical steroids; signs and symptoms improved to grade 1.

Subsequently, the severity of the skin toxicity associated with docetaxel involved treatment-switching to paclitaxel 80mg/m^2^ weekly. After the first week of this treatment neither new toxicities occurred nor pre-existing toxicities increased. After the second week, the patient developed paclitaxel toxicities that included grade 1 diarrhea and grade 1 peripheral neuropathy.

Tolerability of treatment decreased after the third week of paclitaxel, and toxicities included grade 2 asthenia, grade 2 peripheral neuropathy, grade 2 diarrhea, and grade 2 skin and nail toxicity. These toxicities promoted chemotherapy delay and she was treated with gabapentin, loperamide, and oral and topical steroids. The fourth dose of paclitaxel was administered 1 week later; the patient developed the same symptoms and the treatment required a further delay.

**Figure 2:**
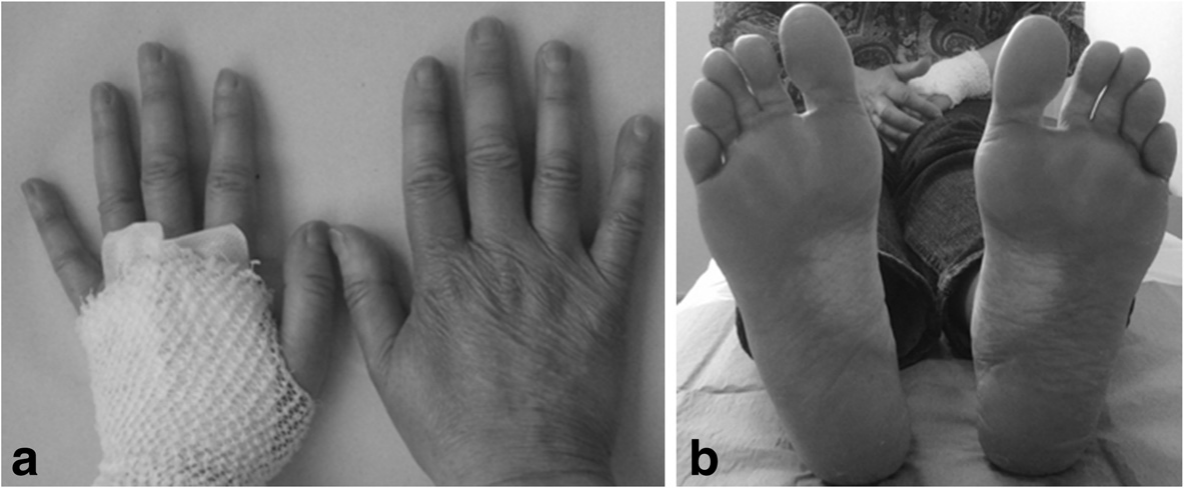
Improvement of skin toxicity during treatment with nanoparticle albumin-bound paclitaxel.

As a consequence of her symptoms and the importance of chemotherapy dose intensity in the neoadjuvant setting, paclitaxel was switched to nab-paclitaxel 100mg/m^2^ weekly. Standard paclitaxel would have required a dose reduction or further delays in treatment (Figure 2).

Tolerability was acceptable with resolution of diarrhea, improvements in skin toxicity and asthenia to grade 1, and persistence of grade 2 neuropathy. She completed the five planned weeks of treatment without delays or dose adjustment.

Post-treatment breast imaging with mammography, ultrasound, and posterior MRI, showed complete response of the retroareolar lesion and partial response of the enlarged intramammary lymph node and axillary lymph nodes.

As a result of this good response, tumorectomy with axillary lymph node removal were performed. Histology showed infiltrating lobular carcinoma measuring 1.5cm with lower anterior and posterior margins involved, and metastases from infiltrating lobular carcinoma with pleomorphic areas and signet ring cells in 20/20 isolated lymph nodes, the largest one measuring 2cm in longest diameter.

As margins were involved, a mastectomy was performed at a second stage and no residual malignancy was found.

## Discussion

The use of taxanes has been associated with a number of skin toxicities. With paclitaxel, administration site reactions are generally rare and mild, ranging from erythema or phlebitis to depigmentation. Paclitaxel is considered irritating rather than vesicant, and lesions from extravasation are usually mild; however, edema, cellulitis, and even necrosis have also been reported. Another type of skin toxicity is the “recall effect”, both in previously irradiated areas and in extravasation sites. Some isolated cases of nail bed ulceration have also been notified. Hypersensitivity reactions, both acute (characterized by skin rash) and delayed (in the form of blisters, diffuse pustular rash with exfoliation, pruritic maculopapular rash, scleroderma-like lesions, or hand-foot syndrome) have been reported [7-10].

With docetaxel treatment, most patients develop some type of skin toxicity, which is usually mild and self-limited. These reactions include hypersensitivity, edema, erythrodysesthesia, erythema multiforme, nail changes, photosensitivity, scleroderma, and subacute cutaneous lupus erythematosus [10-12].

These skin toxicities secondary to taxanes seem to be immune-mediated. Both acute and chronic hypersensitivity reactions may be secondary to the cytotoxic agent itself or to any of its additives. Cremophor® has been reported to be involved not only in hypersensitivity reactions but also in changes in the pharmacokinetics of paclitaxel.

Due to the toxic effects reported, the dose of paclitaxel and docetaxel that can be administered is limited. In chemotherapy, dose intensity is a well-known major determining factor in the prognosis of patients with breast cancer, and even a linear relationship between both was identified [13]. Bonadonna *et al.* reported that the response rate to primary chemotherapy is probably more related to dose intensity than to the chemotherapy regimen itself [14]. The importance of achieving complete response is also well established in the neoadjuvant setting for long-term prognosis, with lower relapse rate and increased overall survival [15].

Nab-paclitaxel has shown good clinical results in first- and further-line therapy of patients with metastatic breast cancer (MBC) and it has also demonstrated considerable activity in taxane-pretreated patients with MBC. Because of the excellent tolerability of nab-paclitaxel in hypersensitivity reactions to docetaxel and paclitaxel, we decided to administer weekly nab-paclitaxel in a patient with locally advanced breast cancer with skin toxicity secondary to delayed hypersensitivity to taxanes. No skin toxicity occurred, which allowed us to complete neoadjuvant treatment.

In 2009, Gradishar *et al*. performed a randomized Phase II study with 302 patients with previously untreated MBC human epidermal growth factor receptor-2 negative patients who either received nab-paclitaxel 300mg/m^2^ every 3 weeks, 100mg/m^2^ weekly or 150mg/m^2^ weekly, or docetaxel 100mg/m^2^ every 3 weeks. The two weekly regimens demonstrated a longer progression-free survival in an independent review, although this was not confirmed by the investigators for the 100mg/m^2^ dose. Peripheral neuropathy was similar, but shorter in duration with nab-paclitaxel [16]. We chose nab-paclitaxel 100mg/m^2^ weekly based on the results of this study and on the patient’s neurotoxicity. In addition, this regimen (nab-paclitaxel 100mg/m^2^ weekly) has demonstrated the same antitumor activity as the 125mg/m^2^ weekly regimen and a more favorable safety profile in patients with MBC that had progressed with previous taxane therapy without severe hypersensitivity reactions reported. Both doses have exerted a good efficacy profile in heavily pretreated taxane-refractory patients [4].

In this case report the pathological response was not good, with persistence of malignancy locally and significant axillary tumor burden. This might have been a result of the tumor biology and its advanced clinical stage at diagnosis.

However, the patient could complete the treatment plan within the scheduled timeframe with acceptable tolerance.

Nab-paclitaxel is expected to have only limited cross-resistance to solvent-based taxanes [4].

## Conclusions

We conclude that nab-paclitaxel is a good therapeutic option for patients with any breast cancer stage who develop taxanes-induced skin toxicity.

## Consent

Written informed consent was obtained from the patient for publication of this case report and any accompanying images. A copy of the written consent is available for review by the Editor-in-Chief of this journal.

## Competing interests

The authors declare that they have no competing interests.

## Authors’ contributions

BCC followed up and managed the patient and drafted the manuscript. VQG drafted the manuscript. CLR managed and controlled drugs. MIPM performed surgery. LCR and NPA managed the patient. SVM and AMM diagnosed the patient. CRC treated and followed up the skin toxicity. MMV drafted the manuscript. All authors read and approved the final manuscript.
